# Protocol of the randomized double blind sham controlled AddVNS study of transcutaneous vagus nerve stimulation mechanisms in depression

**DOI:** 10.1038/s41598-026-42459-2

**Published:** 2026-03-02

**Authors:** Evangelos Kokolakis, Iven-Alex von Mücke-Heim, Julius C. Pape, Norma C. Grandi, Angelika Erhardt, Philipp G. Sämann, Victor Spoormaker, Nadine Gogolla, Elisabeth B. Binder, Peter Falkai

**Affiliations:** 1 Department Clinical Translation, Max Planck Institute of Psychiatry (MPIP), Munich, Germany; 2 Department Genes and Environment, MPIP, Munich, Germany; 3 Department Emotion Research, MPIP, Munich, Germany; 4 Psychiatry Department, Clinical Anxiety Research, University of Würzburg, Würzburg, Germany; 5 Neuroimaging Core Unit, MPIP, Munich, Germany; 6 Department Clinical Translation, RG Translational Neurostimulation, Kraepelinstraße 2-10, 80804 Munich, Germany

**Keywords:** Diseases, Health care, Medical research, Neurology, Neuroscience

## Abstract

Depression is among the most prevalent mental disorders worldwide, carrying one of the highest burden of disease among all mental disorders. While invasive vagus nerve stimulation has been approved for treatment-resistant depression for decades, its clinical use is limited by surgical risks and heterogeneous clinical efficacy. Transcutaneous auricular VNS (tVNS) may offer a non-invasive alternative, but to date it remains experimental due to limited high-quality evidence, unclear biological mechanisms of action, and rudimentary knowledge on optimal stimulation parameters. To address these gaps, we initiated the AddVNS study. The AddVNS study (**Add**-on t**VNS** in depression) is a monocentric, exploratory, prospective, randomized, double-blind, sham-controlled interventional trial conducted at the Max Planck Institute of Psychiatry’s research hospital. Adult patients with a depressive episode (ICD-10: F31–33) were assigned to receive active or sham transcutaneous vagus nerve stimulation (tVNS) in addition to treatment-as-usual (TAU) over a six-week period. Stimulation is administered three times daily (30 to 60 min each), five days per week. A deep phenotyping strategy is applied, including repeated psychophysiological measures (e.g., pupillometry, ECG, photoplethysmography, electrogastrogram) and neuroimaging (structural and functional MRI) at baseline and post-intervention, continuous actigraphy, repeated blood and stool sample acquisition (pre-, mid-, and post-intervention) for multiomic investigation, comprehensive neuropsychology including self-rated personality assessment, and closely monitored clinical evaluations. The patient-reported outcomes are collected weekly, the clinician-rated scales pre-, mid-, and post-intervention. In addition, follow-up self-ratings are obtained at 6 and 12 weeks post-tVNS. The main objective of AddVNS is to improve our understanding of the biological effects elicited by tVNS in depression. By combining rigorous methodology with an extensive and longitudinal multimodal approach, AddVNS represents the most comprehensive investigation of tVNS effects and markers in depression to date. We believe it to significantly advance our mechanistic understanding and subsequently clinical translation of this promising intervention.

**Supplementary Information:**

The online version contains supplementary material available at 10.1038/s41598-026-42459-2.

## Introduction

Depression is the second most prevalent mental disorder worldwide and is associated with the highest burden of disease among all psychiatric disorders^1,2^. The lifetime-prevalence of major depressive disorder (MDD) in Germany is estimated to be 11,6%^3^. MDD is a major risk factor for suicide and is associated with higher risk for somatic diseases and mortality^4–6^ and therefore it is also a vast burden for the healthcare system^7,8^. To treat depression, pharmacotherapy and psychotherapy are the standard of care. Still, response rates are in general moderate: approximately 30% of the patients show a treatment-resistant course of disease^9^. Especially for those patients, neurostimulation treatments like repetitive transcranial magnetic stimulation (rTMS), electroconvulsive therapy (ECT) or vagus nerve stimulation (VNS) are important therapeutic strategies^10,11^.

In Europe, invasive VNS has been approved for the treatment of drug-resistant epilepsy since 1994 and for the treatment of treatment-resistant depression (TRD) since 2001^12,13^. In 2005, invasive VNS was approved by the US Food and Drug Administration (FDA) for the treatment of MDD that has not responded to at least four adequate trials of antidepressants^14^. Invasive VNS requires the surgical implantation of an impulse generator^13^. Because of the associated possible surgical complications as well as high treatment costs, invasive VNS is not often used in the treatment of depression^15^. Transcutaneous auricular VNS (tVNS), on the other hand, is a non-invasive alternative to traditional invasive VNS. During tVNS, a transcutaneous impulse generator stimulates the auricular branch of the vagus nerve (ABVN) using soft electrical currents. The direct neuroanatomical connection of the auricular branch of the vagus nerve to the nuclei of the brainstem is a possible pathway through which tVNS may exert its biological effects^16^. During the last decade, the effects of tVNS have been examined in an increasing number of clinical trials and across many different clinical populations and disorders, with depression being one of the most prominent areas of interest^17^. Studies have jointly suggested that tVNS may be an effective and well tolerated treatment for MDD^18,19^, peripartum depression^20^, and TRD^21^. Evidence for the potential of tVNS to improve MDD comes from meta-analyses, although these analyses included a relatively small number of studies, some of which exhibited methodological limitations^22,23^. Meanwhile, the safety and tolerability of tVNS is well established and it has not been associated with severe adverse effects unlike invasive VNS^15,24^. Potential side effects of tVNS are mild and local symptoms and include, but are not limited to, skin irritation along with non-specific symptoms like transient mild headache or dizziness^15,25^. Along with its tolerability, the enhanced accessibility and ease of application of tVNS, compared to other neurostimulation methods, further contribute to its feasibility^26^.

In the German, but also other national depression treatment guidelines, tVNS is still considered an experimental treatment^11^. Reasons for this categorization come from the relative novelty of the intervention, the limited high-quality evidence from randomized clinical studies, the not yet fully understood biological mechanisms of action^26^, and the limited knowledge about the optimal stimulation parameters^25,27^.

To date, numerous hypotheses have been proposed regarding the biological mechanisms underlying the antidepressant effects of invasive vagus nerve stimulation and, by extension, transcutaneous VNS. The proposed mechanisms include, but are not limited to, changes in functional brain connectivity, modulation of neurotrophins and subsequent increase of neurogenesis, inhibition of systemic inflammation, normalization of the hypothalamus-pituitary-adrenal (HPA) axis, modulation of neurotransmission, and changes in the gut microbiome^26–33^. For these hypotheses, there is only limited or indirect evidence primarily from preclinical studies or from application of VNS in other disorders^26,30^. To date, however, it is unclear which of these mechanisms contribute to the therapeutic effects of VNS in depression. It is thus not surprising that, so far, no reliable bio- or predictive markers exist for tVNS in depression^25^.

To address these issues, we developed and initiated the AddVNS study. The primary objective of the study is to identify biological, psychological, socio-demographic, and clinical biomarkers associated with augmentative tVNS in patients with a depressive episode.

## Study design

**Fig. 1:**
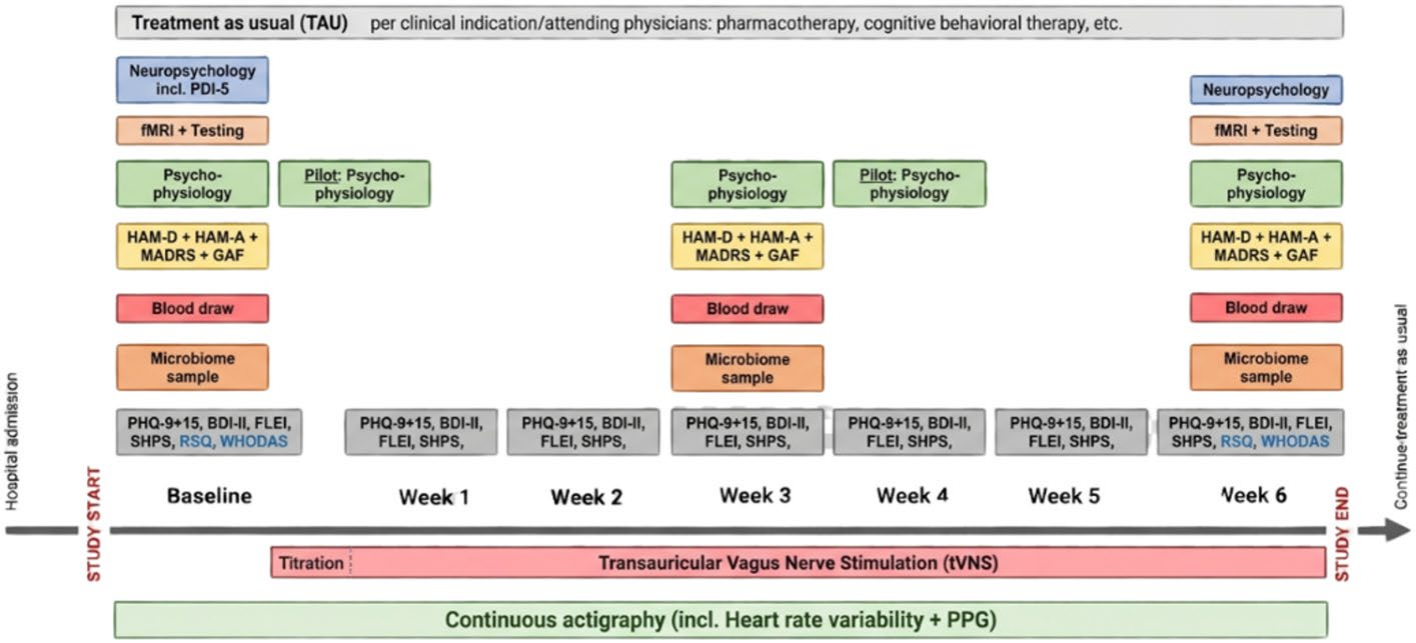
Schematic diagram of participant timeline in the AddVNS study. Patient health questionnaire 9 and 15 (PHQ9 and PHQ15), Beck Depression Inventory II (BDI-II), Snaith-Hamilton-Pleasure-Scale (SHAPS), Rejection Sensitivity Questionnaire (RSQ), World Health Organization Disability Assessment Schedule 2.0 (WHODAS 2.0.), Montgomery–Åsberg Depression Rating Scale (MADRS), Hamilton Rating Scale for Depression with 21 items (HAM-D21), Hamilton Rating Scale for Anxiety (HAM-A), Global Assessment of Functioning (GAF), Pulse plethysmography (PPG), Personality Inventory for DSM-5 Brief form (PDI-5), functional MRI (fMRI). Created with Biorender.com.

The AddVNS study is a monocentric, exploratory, prospective, randomized, double-blind, sham-controlled interventional study with a recruitment period of 3 years. It commenced in 2025 and is conducted at the research hospital of the Max Planck Institute of Psychiatry (MPIP). The study information has been aligned and filled out in the SPIRIT guidelines including a schematic participant timeline diagram (Fig. 1). Also, the research protocol version 1.0, structured in accordance with the Research Ethics Review Committee of the World Health Organization, can be found in the supplement.

Participants receive either tVNS or sham tVNS for 6 weeks in addition to treatment-as-usual (TAU). Psychophysiological assessments, clinical interviews, blood draws, and stool collection are scheduled at baseline as well as 3 and 6 weeks after tVNS start. A functional MRI (fMRI) session as well as an extensive neuropsychological assessment is performed at baseline and after 6 weeks post tVNS start. Additionally, a personality self-report assessment is acquired at baseline. Furthermore, participants are asked to complete self-rating questionnaires on a weekly basis. The optional questionnaire-based follow-ups are inquired 6 and 12 weeks after tVNS end. Study duration thus amounts to 8 weeks plus 12 weeks of follow-up.

**Fig. 2:**
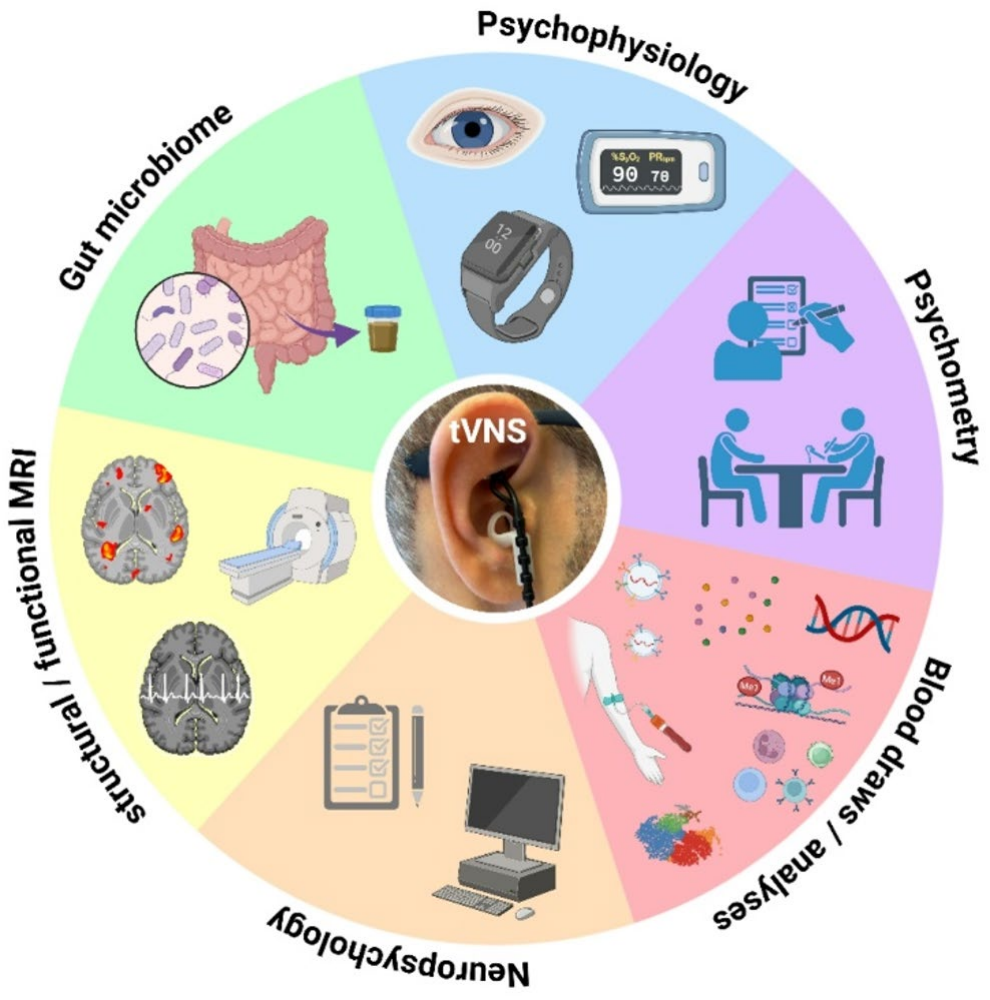
Graphic summary of the AddVNS study’s multiomics approach. Created with Biorender.com.

The study will start with a pilot. Here, two additional psychophysiological measurements (amounts to a total of five measurement time points) will be conducted during tVNS stimulation up to 20 participants after the initial psychophysiological assessment at baseline and following 3 weeks of tVNS application. The objective of this pilot is to examine acute effects of tVNS on psychophysiological parameters, providing first insights into the potential mechanisms of action. Owing to the exploratory and first-in-field nature of this study, patient and public involvement was not included in the study design. The AddVNS study’s multi-omics read-outs are shown in Fig. 2.

### Participants

Only adult patients (≥ 18 years) with current MDD or bipolar disorder with current depression (BD) who are treated at the MPIP’s research hospital (either inpatient or day clinic treatment) and are able and willing to give informed consent can participate in the AddVNS study. Inclusion criteria are: (1) age 18–65 years, legally competent and able to provide informed consent; (2) diagnosis of a depressive episode (MDD or BD) according to ICD-10 or ICD-11; (3) signed informed consent documents for the AddVNS study; (4) signed informed consent and participation in the biobanking project of MPIP (Project No. 338 − 15). Exclusion criteria are: (1) age < 18 years or age > 65 years; (2) pregnancy or planning to get pregnant during the study period, breastfeeding; (3) legal supervision; (4) pervasive developmental disorders and/or intellectual disability; (5) acute substance abuse (e.g., alcohol, prescription or illicit drugs); (6) severe neurological disease; (7) anatomical or technical impossibility of tVNS administration (e.g., microtia, anotia, or history of vagotomy); (8) current treatment with an established neurostimulation method (e.g., ECT, rTMS, VNS); (9) metallic foreign bodies, implanted intracranial devices or cerebral shunts; (10) severe general illness (e.g., relevant anemia requiring transfusion, high-grade cardiac arrhythmia, severe cardiomyopathy); (11) active implants (e.g., cochlear implant, pacemaker, implantable cardioverter-defibrillator).

### Randomization and blinding

After inclusion, participants are randomized to verum or sham tVNS. Participants are blinded to their study arm, since the verum and sham electrode look alike and the weekly titration was performed in both groups. Investigators performing study assessment are also blinded to the study arm, except for study personnel responsible for the stimulation parameter settings and weekly titration. A block-wise randomization approach was chosen^34^. This design was selected to reduce the likelihood that participants in different intervention arms would share information about their stimulation-related experiences or sensations. Participants within a block receive the same intervention. Block sequence and size was based on an allocation list. A new block is started after the study participation for the patients of the previous is completed. Blinding integrity was assessed during the final week of the sham/verum tVNS by asking participants to indicate which treatment arm they believed they had been assigned to.

### Intervention

Participants receive either tVNS or sham tVNS for a period of 6 weeks. Both interventions are administered as an adjunct to TAU, which is provided by the participants’ attending physicians and psychologists according to clinical expertise and guidelines. Both tVNS and sham tVNS intervention are carried out three times a day (morning, noon, evening) from Monday to Friday. Each of the three daily stimulation sessions lasts 30 to 60 min, depending on the individual tolerance (= individual subjective comfort and tolerability of stimulation) of the participant. Flexibility in stimulation duration was permitted to optimize adherence by accommodating individual tolerability, preferences, perceived autonomy, and comfort.

The stimulation is performed with the device tVNS E ^®^ (tVNS Technologies GmbH, Erlangen, Germany). The device is CE certificated and approved as Class IIa medical device under the Medical Device Regulation of the European Union. The device consists of a stimulator the size of a small cell phone and an ear electrode that is placed at the stimulation site.

The stimulation protocol was developed considering earlier tVNS studies and the mandatory sections of the device’s instruction manual^20,21,35^. The stimulation site is the cymba concha of the left ear, since the latter is thought to be exclusively innervated by the vagus nerve^36^. Thus, the left vagus nerve is stimulated (similar to invasive VNS), which is thought to reduce the risk of bradycardia since the right vagus primarily innervates the sinoatrial node of the heart, although human studies are still needed to confirm this distinction and its clinical relevance^37^. Nonetheless, right-sided or even bilateral tVNS has been shown to be safe^15^. Prior to the actual stimulation, stimulation intensity (electrical current) is determined by titration. Here, the highest level (mA) tolerated by the patient, that is the value between the individual perception- and pain threshold, is used for the longitudinal tVNS stimulation. During weekly titration sessions, stimulation intensity was adjusted beginning at the lowest level (0.1 mA) and increased in 0.1 mA increments until a clearly perceptible yet comfortable sensation was achieved, without pain, up to a maximum of 5 mA. Electrical current of the tVNS E ^®^ can be adjusted between 0.1 and 5.0 mA. Other stimulation parameters are set to the device’s default settings: biphasic stimulation, frequency 25 Hz, pulse-pause ratio 28 s : 32 s.

During the first tVNS stimulation session, study personnel provide detailed instructions on how to use the device in person. In the following sessions, participants perform the thrice daily stimulations by themselves and can reach out to study personnel if questions arise. Any adjustments to the current intensity, including titration during the intervention period, are performed by study personnel only. If a participant intentionally alters stimulation parameters including localization of the ear-electrode in a way that endangers either blinding or the study objective, the individual will be excluded from the study. In addition, regular check-ups are performed by study personnel to prevent errors during the stimulation.

Sham tVNS is performed identical to tVNS, with the only exception that the sham tVNS is conducted with no current using the manufacturer’s tVNS E ^®^ sham electrode. Visually, the sham electrode cannot be distinguished from a regular stimulation electrode. This approach was chosen to maximize discrimination between specific neurostimulatory effects and non-specific contextual or placebo effects, while still matching key elements of intervention.

The time and duration of each stimulation session is documented by the patient. Additionally, the app (*tVNS® Patient*) is used to retrieve, track, and double-check stimulation parameters. This systematic documentation enables dose–response analyses. To prevent unblinding, only investigators responsible for stimulation have access to the app.

### Measures

Sociodemographic data (e.g., age, sex and gender, occupation) and medical information (e.g., patient-, family-, and drug anamnesis, medication history and current medication, results of clinical diagnostics and clinical tests such as ECG and EEG) are extracted from patient records.

### Psychophysiological assessment and actigraphy

Psychophysiological measurements are conducted at baseline, after 3 weeks of intervention and at the end of the 6-week intervention period. These three measurements are acquired without simultaneous tVNS. In the pilot (*n* = 20), additional psychophysiological measurements will be additionally conducted immediately after starting the morning tVNS stimulation session at baseline and following 3 weeks of tVNS application. The measures encompass an N-Back task and a reward task. In the N-back task, which evaluates the working memory capacity, a sequence of stimuli is presented to the subjects, who indicate through pressing a button, if the current stimulus matches a stimulus presented N steps earlier in the sequence^38^. In AddVNS, the visual stimuli are capital letters (the consonants B, C, D, G, P, T, W). The reward task has been reported in earlier studies of our institute^39^. The variant that will be used in the AddVNS study comprises four conditions: a potentially rewarding response condition with a reward stimulus, a neutral response condition (neutral stimulus), a potential loss condition and a control condition without a response requirement (stimulus without response). After the presentation of the stimuli and a brief flash of light, the subjects must respond by pressing a button. If they respond fast enough, they will receive a financial reward (1 €) or a non-monetary reward (a green checkmark symbol). If the reaction is too slow, a red cross appears, which is followed by a financial loss (-1 €) in the loss condition. In the control condition, no flash is presented and therefore no reaction is required. An adaptive algorithm ensures that participants are successful in around 50% of reward trials. Pupil measurements and reaction times are recorded during the reward task. Furthermore, short measurements (e.g. resting state pupillometry, pupillometry during five separate light flashes, word-pairs association task) will be used as control conditions.

For the psychophysiology measurements, we use Biopac’s MP150 System (Goleta CA, USA, biopac.com) in our psychophysiology lab. Pupillometry is assessed with an inhouse Unity-based presentation environment (stimuli are presented on a screen in an Office) for an HTC Vive Pro Eye system with Tobii Eye-Tracking, with a sampling rate of 120 Hz (HTC Corporation, Taoyuan, Taiwan). During the psychophysiological measurements in our psychophysiology lab, the following parameters are assessed using the MP150 system with Bionomadix Wireless units: a 3-lead ECG with one electrode under the right collar bone, one under the left collar bone and one above the left-front side of the hip (LEAD II), respiration with a respiratory belt on the lowest part of the diaphragm, pulse plethysmography acquired on the left middle finger and a 3-lead electrogastrogram on the stomach area. The activity pattern and continuous physiology of participants over the 6-week intervention including the repeated psychophysiology lab session are measured using a wrist-worn actigraph (ActiGraph LEAP, Ametris, Pensacola FL), which provides continuous measures of activity, heart rate, oxygen saturation, and skin temperature, among others. The heart rate signal forms the basis for further processing, with the RR-intervals as inputs for segmented, sliding-window-based heart rate variability analyses (spectral metrics, statistical metrics, and respiratory-sinus arrhythmia-based metrics).

For pupillometry, the primary focus of the analyses will be on measures summarizing the pupillary light reflex (e.g., constriction velocity, amplitude), pupillary unrest (e.g., mean and variance metrics) during a 6-minute resting-state, mean pupil values during working memory blocks, and pupil responses during reward anticipation as in^40,41^. Pupillometric data will be pre-processed with linear blink-interpolation, smoothing and z-transformation, among others^40,41^, with in-house scripts in Matlab 2025 (The Mathworks Inc., Natick, Massachusetts, 2025). EGG will be spectrally analyzed according to consecutive 256s epochs, with 192s overlap according to the *Handbook of Psychophysiology*,* Chap. 12 – Levine et al.*^42^.

### MRI scans

Two identical MRI sessions are acquired. The first session is scheduled at baseline, the second one after the 6-week of tVNS. The elements of the MRI assessment are shared with the imaging protocols of our institute’s deep-phenotyping study BeCOME (Project No. 350 − 14)^43^. MRI protocols include an anatomical T1-weighted sequence, a diffusion tensor imaging (DTI) and an axial fluid-attenuated-inversion-recovery (FLAIR) sequence. Moreover, resting state functional MRI (rs-fMRI) and the following specific fMRI sequences and tasks are obtained:

(i) Neuromelanin (NM)-sensitive MRI: this sequence will be used to measure the functional and structural status of the locus coeruleus, the substantia nigra and the area tegmentalis ventralis (VTA). It is an established method used in the research of the noradrenergic and dopaminergic neurotransmission^44–47^. The NM-sensitive sequence will also allow to precisely localise these brainstem structures for the functional analyses.
(ii) Brainstem-sensitive fMRI-BOLD sequences: This is a sequence for fMRI-BOLD time series aligned to methodological recommendations of a sequence and post-processing comparison for brainstem imaging^48^. It allows, in combination with appropriate post-processing, the investigation of the functional relationship between individual brainstem nuclei^48^.
(iii) Reward anticipation task: This established task^43^ allows the quantification of three key circuits during reward anticipation (early activation of the ventral striatum, later activation of the salience network and deactivation of the default mode network). As described earlier for the reward task during psychophysiological measurements, a further stimulus type, which results in a small loss of money if performance is too low, will be added to the three established stimuli (possibility of winning money, verbal feedback, no possibility of winning). Overall, small symbolic play money sums with maximum total gains of 1–5 € will be used. The dynamic aspects of the task have been studied using combined fMRI/pupillometry experiments in healthy subjects^39^ and patients with major depressive disorder^49^. To allow for flexible secondary statistical methods, we will define between 2 and 5 regions-of-interest per network from large independent samples that have undergone the same task (e.g.^43^.
(iv) Short imaging stress test (short-IST): This task is a reduced version of an imaging stress test^43,50^ that combines arithmetic calculation with (negatively feedback) evaluation. This reduced 10’ version presents 1-minute-episodes of arithmetic calculations with mild evaluation alternating with 40 s of pausing, causing marked autonomous nervous system (ANS) response that can be mapped to central ANS perception or regulation processes^51^.

rs-fMRI and reward-fMRI are carried out with simultaneous eye tracking and pupillometry (EyeLink 1000 Plus System with long range (MR) Mount, SR Research). Furthermore, heart and respiratory rate are recorded throughout the MRI scan using pulse plethysmography and a respiratory belt. No MRI contrast agent is injected.

### Blood-based multiomics

The collected blood samples will be used for genome-wide genotyping and epigenetic/DNA methylation analysis as well as mRNA sequencing, analysis of neuron-derived exosomes, cytokine and immune metabolite analysis, immune cell phenotyping and analysis of metabolic and endocrine markers. For more details on blood-based analyses, we refer the interested reader to the published protocol of DetECT study^52^, which proposes the same multiomics measures.

### Stool samples

Stool samples are collected at baseline and after 6 weeks of verum or sham tVNS using the Bunny Wipe Fecal Sample Collector System (Zymo Research). If tolerated by a participant, additional sample collection after three weeks of tVNS is performed. Stool samples will undergo shotgun metagenomic sequencing, as done in related studies, to enable taxonomic and functional characterization of the gut microbiome over time.

### Psychometric measures

Self-rating questionnaires along with clinician-rating scales are used to systematically evaluate the patients’ mental and physical condition in detail and to assess the effects of tVNS over the course of treatment. All questionnaires and rating scales used in the AddVNS study are well established and standardized instruments in clinical practice and research. Only German versions of the questionnaires and rating scales are used.

Participants are asked to fill out the following questionnaires once a week: patient health questionnaire (PHQ) 9^53–55^ and 15^53,56^, Beck Depression Inventory II (BDI-II)^57^, the Snaith-Hamilton-Pleasure-Scale (SHAPS-D)^58,59^ and the Questionnaire for complaints of cognitive disturbances (FLEI)^60^. The PHQ-9 with nine items, the BDI-II with 21 items and the SHAPS-D with 14 items are self-rated scales used widely in the treatment and research of depression. The PHQ-15 with will be used to assess the physical symptoms of the participants. Free fields were added to the PHQ-15 for the patients to report side effects or physical complaints related or attributed to tVNS. The FLEI World Health Organization Disability Assessment Schedule 2.0 (WHODAS 2.0.) with 35 questions is used to evaluate the patients’ neurocognitive symptoms. At baseline and after the 6-week intervention, participants also fill out the Rejection Sensitivity Questionnaire (RSQ)^61,62^ and the World Health Organization Disability Assessment Schedule 2.0 (WHODAS 2.0.) questionnaire^63^. The German modified version of RSQ with 20 items^62^ is a measure of rejection sensitivity (RS) to explore the role of RS in the context of tVNS in depressive disorders. The WHODAS 2.0. questionnaire is a self-rating instrument with 36 items for the assessment of health, functioning, disability and health-related quality of life which can be used across all diseases^63^. Questionnaires are available as paper-pencil printed version or electronically (QR-code or email link or tablet computer, all via a secure study platform).

Clinical interviews are conducted at baseline, after 3 weeks of intervention and after 6 weeks of intervention. During these interviews, trained study personnel obtains the following scales: Montgomery–Åsberg Depression Rating Scale (MADRS)^64,65^, Hamilton Rating Scale for Depression with 21 items (HAM-D21)^66–68^ and Hamilton Rating Scale for Anxiety (HAM-A)^69^. The Global Assessment of Functioning (GAF)^70,71^ will also be obtained using information provided by the patient`s clinical ward team at the same time points. The MADRS (10 items) and the HAM-D21 are used to assess the severity of the present depressive episode. The GAF, which is a compound scale derived from the DMS-IV that rates global socioemotional impairment of patients with mental disorders, is used as a functionality proxy.

### Neuropsychological assessment and personality assessment

Neuropsychological assessment is carried out at baseline and after the 6-week intervention using the Cognitive Basic Assessment Test set (COGBAT)^72^ as well as the “Mehrfachwahl-Wortschatz-Intelligenztest” (MWT-B)^73^ and an episodic memory test^74^. The COGBAT was administered using the standardized software and hardware provided by the test distributor (Schuhfried). The MWT-B and the episodic memory test are administered in their respective standardized printed formats in accordance with the publishers’ guidelines.

Personality assessment is performed at baseline using the German short version of the Personality Inventory for DSM-5 (PID-5-BF + M) and the German short version of the Level of Personality Functioning Scale-Brief Form 2.0 (LPFS-BF 2.0)^75–77^. Both questionnaires can be filled out quickly by patients and allow a reliable assessment of personality traits. In the AddVNS study, PID-5-BF + M and LPFS-BF 2.0 will be used to investigate the role of personality traits in the depressive episode in the content of the biological effects of tVNS.

### Follow-up

To assess the long-term effects of tVNS, including the lasting self-reported clinical effects in e.g. the home environment after discharge from clinical care, we ask the participants to fill out self-rating questionnaires 6 and 12 weeks after the end of the intervention. At each of these two points, the questionnaires BDI-II, PHQ-9, PHQ-15, SHAPS-D, FLEI and WHODAS 2.0. are obtained. In case the participants have been discharged prior to the follow-up, they will receive the paper-pencil questionnaires (by mail) or be asked to complete them electronically after receiving a link via email, in accordance with our data protection and privacy policy.

### Sample size calculation

Earlier tVNS studies with a design similar to AddVNS, which have focused on clinical outcomes rather than biological ones, have shown low to moderate effect sizes^18,19,23,35^. Moreover, depression is a complex and multifactorial disease, which emerges through the long-time interaction between genetic predisposition and environmental factors^78^. Since no prior multi-omics studies on tVNS effects are available, the sample size calculation was based on the average effect sizes reported in existing clinical tVNS studies. This approach assumes that clinical efficacy reflects the aggregate downstream effects of multiple interacting biological pathways underlying tVNS and can therefore serve as a pragmatic proxy of expected average effect size of omics domains in the absence of direct multi-omics effect size estimates. Such an approach can be considered contemporary and appropriate for early-phase or exploratory multi-omics trials^52,79,80^. In addition, the AddVNS study incorporates an internal pilot phase. Should pilot data unexpectedly indicate that the main assumption regarding effect size is significantly inaccurate, sample size estimates will be updated prior to completion of recruitment, in line with established methodological recommendations^81,82^. Using a G-power calculation tool^83^, we estimated a sample size of *n* = 86 (ANOVA with repeated measures, 2 groups, effect size Cohen’s f = 0.25, significance level a = 0.05, power = 0.95, allocation in study arms 1:1, correlation between repeated measures = 0.3).

Based on prior experience with stimulation studies conducted at our clinic^52^, the AddVNS study anticipates a study discontinuation rate (including loss to follow-up) of approximately 30%. To account for this, we plan to recruit up to *n* = 112 participants, ensuring that at least *n* = 86 complete datasets are available for statistically meaningful analyses in accordance with the sample size calculation. Data from participants who discontinue the study will be included in the final analyses under an intention-to-treat framework. In addition, if more than 10% of data are missing, missing values will be handled using established imputation methods and the results will be compared with those obtained from the intention-to-treat analyses. Using heart rate variability-based stratification, among other psychophysiological and fMRI measured (e.g., pupil size), subgroup analyses will be performed.

### Main hypotheses and statistical analyses

The focus of the statistical analyses in the AddVNS study is to examine the longitudinal changes between the two study arms (tVNS vs. sham tVNS) at the level of neurobiological readouts. Our aim is to identify parameter patterns that allow direct or indirect conclusions *primarily* about the biological effects of tVNS and *secondarily*, its clinical effects. Age, sex, socioeconomic status, BMI, smoking status, and pharmacological treatment (psychiatric and otherwise) as well as comorbidities will be integrated as covariates. Analyses will be performed regularly (e.g., after the pilot phase) as well as at the study’s end.

At the **neuroimaging level**, given the neuroanatomy of tVNS^16^ several functional readouts will be used for the study of tVNS effects over 6 weeks, contrasted against sham treatment. The general underlying hypothesis is that the 6-week stimulation course produces midterm-lasting functional effects that could underlie parallel or future clinical effects.

(i) Functional reward system response changes that comprise the VTA and nucleus accumbens / ventral striatum, and possibly also the locus coeruleus itself, can be detected by the monetary delay reward task^43^.
(ii) Exploratively, comparisons of task-free brainstem fMRI data will be performed after brainstem-focused preprocessing, based on reports on short term effects in downstream targets of vagal afferents including the nucleus of the solitary tract, the substantia nigra, subthalamic nucleus and periaqueductal gray^84–87^. Brainstem intrinsic connectivity changes will be explored by simple-ROI-by-ROI cross correlation analysis, and emerging focal brainstem effects can be followed up by seed-to-brain analyses.
(iii) Using the short-IST, we investigate if the central cortical mapping of sympathetic-parasympathetic balance – as monitored by the task/rest switches – change over 6 weeks of treatment. Established parametric modulation techniques will be used here to fuse autonomous signals (e.g., heart rate) with the fMRI time series analysis^51^.
(iv) NM-sensitive imaging: Direct quantification of the LC (and other structures with a NM-positive signal such as the VTA and the substantia nigra) will be used for baseline/follow-up comparisons after adequate preprocessing to evaluate potential differences in noradrenergic or dopaminergic trafficking. To date, it is not known if mid-term effects (follow-up measurement days after the last tVNS/sham stimulation) can be captured using this technique.

### Study objectives

The primary hypothesis of our study is that tVNS induces biological and psychophysiological changes in patients with depression, and that the magnitude of these changes is associated with the antidepressant effects of tVNS. All collected data will be used to evaluate whether beneficial tVNS effects (in addition to TAU) can be predicted. Results will be presented on conferences and published in peer-reviewed journals and, if appropriate, on the Max-Planck-Institutes website in plain language for relevant groups (e.g., participants, physicians).

The main objective of the study is therefore to identify neuroimaging and psychophysiological changes associated with tVNS. The study’s secondary objective is the exploratory investigation of individual markers or clusters of clinical, psychometric, and biological markers which may relate to the course of tVNS in depressive patients receiving TAU (multiomics). Moreover, these parameters as well as the course of treatment (e.g. effects and side effects) will be correlated with the tVNS stimulation paradigm, such as current intensity and daily stimulation duration. The collected data will be used to develop predictive models aiming to identify factors associated with beneficial effects of tVNS in patients with depression.

### Ethics

The study has been approved by the ethics committee of the Ludwig-Maximilians-University Munich (project registration number: 24–0985). The study is registered in the U.S. National Institute of Health (NIH) clinical study register www.clinicalTrials.gov (ID: NCT07022171; registration date: 16/05/2025). The study was developed and is conducted in accordance with the Declaration of Helsinki and all relevant national and international regulations.

All participants must provide a signed informed consent obtained in person by a trained study physician. Participants are further informed that they can withdraw from the study at any time without consequences or disadvantages for their clinical treatment. Participation in the study is voluntary and not compensated or paid. Protocol modifications will be reviwed by the ethics committee prior to implementation. Protocol and informed consent versions are documented on the respective documents.

## Discussion

In summary, the AddVNS study will investigate the change of multimodal biological markers during adjuvant tVNS and the relationship to clinical course and change in depressive symptoms during adjuvant tVNS in patients with MDD. This will be achieved by leveraging the sham-controlled design.

To date, our biological and clinical understanding of tVNS in depression is limited and reliable biomarkers are lacking^25^. Still, functional MRI studies have already produced promising results: tVNS can modulate the functional connectivity (FC) in the striatum and in the default mode network (DMN) in patients with MDD^88,89^. Moreover, an increased activity of the left insular cortex was observed in depressed patients receiving auricular tVNS with partial predictive value for treatment success^90^. It is also worth mentioning that modulation of the locus coeruleus (LC) through tVNS has been shown in patients suffering from migraine^91^. Despite these promising findings, functional neuroimaging studies still face significant challenges, which are to some extent the result of the specific technical requirements needed to reliably detect activation of brainstem nuclei, such as high spatial resolution or advanced postprocessing methods^25,92^. To address this issue, we have incorporated an NM-sensitive sequence into our MRI protocol, which enables a reliable visualization of the LC, along with brainstem-sensitive fMRI-BOLD sequences. This approach overlaps with recommendations regarding the utilization of fMRI in tVNS trials^25^. Another novel aspect of the MRI protocol used here is the inclusion of two fMRI tasks that allow for insights into depression-circuit specific effects of tVNS. First, an established monetary incentive delay task by its VTA and ventral striatum / nucleus accumbens activation provides a functional readout of reward anticipation processes that are disturbed in MDD^93^. Novel results from high resolution imaging suggest a closer convolution between the LC-NE-system (also see below) and the substantia nigra/VTA system than long assumed^94^. Further, the task produces robust default mode deactivation and salience network activation, interlinked with pupil responses, that can be analyzed as previously reported in healthy subjects and MDD patients^39,40,95^. Second, we employ a short version of an imaging stress test (short-IST) to calculate individual maps of the central/peripheral ANS interaction. Using the full version of stress test, ANS responses modulated BOLD signals in areas of the default mode network and salience network (including the insula), but also in hubs of stress response regulation such as the anterior hippocampus and amygdala^51^. The shorter version used here is also explored because of its translatability to clinical routine protocols.

The LC is a brainstem nucleus that serves as the primary source of noradrenaline (NE) in the brain^96^. The LC-NE system is relevant for a variety of psychiatric and neurological disorders, such as Parkinson’s disease^97,98^, Alzheimer’s disease^98^ and depression^99^. Since the ABNV projects to the Nucleus tractus solitarius (NTS), which in turn projects to the LC and many other brain regions including amygdala and insula, the NTS and LC are considered main target sites of tVNS^16^. Pupillometry is a precise and sensitive measurement method for mapping the neuronal activity of the LC^100^. However, studies on the effects of tVNS on pupil alteration have so far yielded heterogeneous results and focused on healthy individuals^101–103^. AddVNS is, to the best of our knowledge, among the first studies that examine the effects of 6 weeks of thrice daily tVNS on pupil dilation dynamics in depressed patients. Besides pupillometry, the psychophysiological assessment during the AddVNS study also includes heart rate variability, which is viewed as a biomarker candidate for tVNS^101^, and the evaluation of gastric mobility using an electrogastrogram. The latter represents a potentially relevant parameter for VNS in general, due to the vagus nerve’s intense innervation of the stomach. However, it has not yet been utilized in the context of tVNS in depression.

Although the identification of psychophysiological and imaging biomarkers for tVNS is the main goal of the current study, other biomarker candidates, with a specific focus on blood-based multiomics will be analyzed. The bidirectional relationship between depression and inflammation has been well established^104^, with studies suggesting that approximately 30% of depressive patients show mild signs of systemic inflammation^105,106^. At the same time, animal studies have shown that the vagus nerve exhibits an anti-inflammatory function as part of the *cholinergic anti-inflammatory pathway* (CAP)^107^. Nevertheless, two recent meta-analyses have found no clear indication for the anti-inflammatory effect of VNS in humans^30,108^. A major limiting factor of these findings is the great heterogeneity of the reviewed and analyzed studies, particularly regarding patient populations and control conditions^108^. The above-mentioned meta-analyses also did not include studies utilizing tVNS in depression^30,108^. Thus, the effects of tVNS on systemic inflammation in depression should be considered undefined, for the time being. Here, the AddVNS study may help bridge this knowledge gap. Moreover, the multiomics approach includes the analysis of both purinergic signaling pathways and neural cell-derived exosomes. These parameters have been repeatedly linked to neuropsychiatric disorders including depression and course of disease and are therefore emerging biomarker candidates^109–113^. They have, however, not been examined in the context of tVNS in depression. The latter also applies to gut microbiome. The vagus nerve is a prominent part of the microbiota-gut-brain axis, which is more and more considered to play an important role in the pathogenesis of depressive disorders^114^. Many mechanisms, through which the microbiota-gut-brain axis can modulate the onset and course of depression, have been proposed, such as immunomodulation, modulation of the HPA axis and modulation of neurotransmitters^28,115^. Nevertheless, the specific neuronal networks involved in the microbiota-gut-brain axis as well as the exact mechanisms of interaction between the vagus nerve, the microbiota and the immune system remain to be elucidated^115,116^. The AddVNS study is, to the extent of our knowledge, the first study to examine the effects of tVNS on the gut microbiome of depressive patients.

Another strength of the presented study is the detailed documentation of the tVNS procedure, including stimulation parameters such as total stimulation intensity, duration, and cumulative electrical charge delivered along with possible adverse events. This allows us to overcome a key drawback of earlier studies, which inconsistently reported tVNS stimulation parameters and side effects^17,24,25,101^. Furthermore, in contrast to our study, many investigations of the biological mechanisms or potential biomarkers of tVNS have examined healthy individuals or lacked a valid control group^101^. Even when a control condition was included, many studies likely suffered from functional unblinding. We aim to circumvent the latter by implementing an elaborate blinding procedure including a sham tVNS electrode indistinguishable from the real one as well as a block-wise randomization strategy to minimize the risk of participants discussing the physical sensations experienced during stimulation across treatment groups. Creating an appropriate sham tVNS protocol is a noted challenge^92^ and does apply to our as well as all other tVNS studies.

The AddVNS study may advance our understanding of the biological effects of tVNS in depression. Ultimately, data and results will determine the extent to which AddVNS can translate into clinically meaningful benefits, guide personalized treatment strategies, and inform future large-scale trials.

## Supplementary Information

Below is the link to the electronic supplementary material.

Supplementary Material 1

## Data Availability

The datasets used and/or analyzed during the current study will be made available from the corresponding authors upon reasonable request, contingent on participants informed-consent approval for respective data sharing and only in compliance with the applicable regulations.
