# Supplementary material for: Protocol of the randomized double blind sham controlled AddVNS study of transcutaneous vagus nerve stimulation mechanisms in depression
Source: Sci Rep. 2026 Mar 2;16:8149. doi: 10.1038/s41598-026-42459-2 (PMC12960843; doi:10.1038/s41598-026-42459-2)
Supplement: Supplementary file 1 — Supplementary Material 1 [file 41598_2026_42459_MOESM1_ESM.pdf]

# - Research protocol of the AddVNS-Study Version 1.0 -

in accordance with the recommended format

of the Research Ethics Review Committee

of the World Health Organization

Project summary

Invasive vagus nerve stimulation (VNS) is an approved treatment of treatment-resistant depression (TRD) in Europe and in USA. Because of the associated possible surgical complications as well as side effects, invasive VNS is applied limitedly in the treatment of depression. Transcutaneous auricular VNS (tVNS), on the other hand, is a non-invasive alternative to traditional invasive VNS. tVNS is still considered an experimental treatment for depression. This is due to the limited high-quality evidence from randomized clinical studies, the not yet fully understood biological mechanisms of action, along with overall limited knowledge about the optimal stimulation parameters. To address these issues, the AddVNS study was initiated. The AddVNS study intends to recruit n=86 patients of the Max Planck Institute of Psychiatry with depression. The patients participating in the AddVNS study are going to receive either tVNS or sham tVNS for a period of 6 weeks. The primary objective of the study is to identify biological, psychological, socio-economic, and clinical biomarkers associated with treatment progression and response to treatment in patients with depression undergoing tVNS. To achieve this, an exploratory design with an assessment of many different parameters including psychophysiology, imaging, blood-based multi-omics, microbiome, psychometrics and neuropsychology will be used.

General information

<u>Protocol title:</u>
The AddVNS-study: Investigation of the physiological and molecular mechanisms of action of transcutaneous auricular vagus nerve stimulation in people with depression."

Registration: ClincalTrials.gov - NCT07022171; Registration date: 15.06.2025

<u>Name and address of the sponsor/funder</u>: none (institutional budget)

Name and title of the investigator(s) who is (are) responsible for conducting the research, and the address and telephone number(s) of the research site(s), including responsibilities of each

Study site:	Max Planck Institute of Psychiatry<br>Clinic for Psychiatry, Psychotherapy,<br>Psychosomatics and Neurology<br>Center for Neurosciences<br>Kraepelinstraße 2–10, 80804 Munich
Central analysis unit:	Max Planck Institute of Psychiatry<br>Kraepelinstraße 2–10, 80804 Munich

Approvals ethics committees: obtained (University of Munich, LMU, ID: 24-0985, 18.03.25)

Lead Investigator (PI)	Prof. Dr. med. Peter Falkai<br>Max Planck Institute of Psychiatry<br>Kraepelinstraße 2-10, 80804 Munich	
	Phone	+49 (0) 89-30622-1000
	Mail	Peter.Falkai@med.uni-muenchen.de

Study physicians and involved researchers:

Prof. Dr. Dr. Elisabeth Binder	Director, Investigator
Dr. med. Evangelos Kokolakis	Study physician / Investigator, Coordinator
Dr. med. Iven von Mücke-Heim, MSc	Study physician / Investigator, Coordinator Dr.
Prof. Dr. med. Angelika Erhardt	Study physician / Investigator
Dr. med. Philipp Sämann	Study physician / Investigator
Dr. Victor Spoormaker	Researcher

Rationale & background information

Depression is one of the most prevalent mental disorders worldwide and is associated with the highest burden of disease among all psychiatric disorders. Invasive vagus nerve stimulation (VNS) has been approved for the treatment of treatment-resistant depression (TRD). However, invasive VNS is applied limitedly in the treatment of depression due to the associated possible surgical complications and side effects. Transcutaneous auricular VNS (tVNS), on the other hand, is a non-invasive alternative to invasive VNS. During the last decade, the effects of tVNS have been examined in an increasing number of clinical trials across many different disorders, with depression being one of the most prominent areas of interest. Nevertheless, tVNS is still considered an experimental treatment for depression. Reasons for this categorization come from the limited high-quality evidence from randomized clinical studies, the not yet fully understood biological mechanisms of action, along with overall limited knowledge about the optimal stimulation parameters. To address these issues, we initiated the AddVNS study. The primary objective of the study is to identify biomarkers of tVNS and to better understand its biological mechanisms of action in the treatment of depression.

We postulate that the biological effects of tVNS, similar to invasive VNS, are likely based on an interaction of various short- and long-term effects of vagus nerve stimulation, along with secondary modulatory processes. For the individual mechanisms and hypotheses, there is generally little or only indirect evidence—derived from anatomical studies of the vagus nerve, from related applications of vagus nerve stimulation in other disorders, or from preclinical research [1, 2]. The proposed mechanisms of tVNS can be summarized as follows: alterations in functional connectivity; modulation of neurotrophins such as BDNF; enhancement of neurogenesis and neuroplasticity; reduction of blood–brain barrier permeability; inhibition of central and peripheral inflammation; regulation or normalization of the hypothalamic–pituitary–adrenal axis; modulation of neurotransmitter release and neurotransmission; and changes in gut microbiota composition [1-9].

Which of these processes dominate in individual patients, or which are decisive for therapeutic success, remains unclear. Moreover, although insufficiently researched, it is plausible given the clinical and biological heterogeneity of depression that specific subpopulations of patients, defined by certain characteristics, benefit more from adjunctive tVNS than others.

Study goals and objectives

Primary project objective: The main aim of the study is to identify psychophysiological and imaging parameters that are associated with the clinical course under tVNS compared with a control condition (sham tVNS). In addition, these markers—together with the clinical-psychological course (including efficacy and side-effect profile)—will be correlated with the tVNS stimulation paradigm (e.g., titration procedure, daily stimulation duration, frequency, amplitude). By integrating all measurements, it could be determined whether certain parameter constellations (biomarkers) influence the clinical course of tVNS in individual patients, and which biological processes underlie these associations.

Secondary project objectives: Exploratory scientific analysis of individual markers or clusters of biological (multi-omics) and psychological parameters in cross-sectional and longitudinal comparisons of tVNS versus sham stimulation. The collected data are also expected to support the development of new hypotheses regarding the specific mechanisms by which tVNS exerts its effects in depressive disorders.

Study design

The AddVNS study is a single-center, exploratory, prospective, controlled interventional study with a planned observation period of approximately eight weeks plus follow-up (i.e., around 6 and 12 weeks after the end of stimulation) for each participant. Study procedures and recruitment are conducted at and by the Max Planck Institute of Psychiatry. Recruitment is performed at the Max Planck of Psychiatry's research clinic. After AddVNS written informed consent is obtained, participants are randomized into two study arms (tVNS = stimulation = intervention group vs. sham tVNS = sham stimulation = control group). Participants are blinded to their group assignment. Investigators are also blinded to participants' group allocation, with the exception of the study physicians responsible for administering the stimulation (double-blind design). The measurements described in detail below in the methods section (psychophysiology, MRI, blood sampling, stool sample collection, questionnaire-based psychometrics, neuropsychology including personality assessment, and follow-up) are conducted in both study arms.

Methodology

The AddVNS is a monocentric, exploratory-prospective, randomized, double-blind, sham-controlled interventional study that recruits adult and legally competent patients with current MDD or bipolar disorder with current depression who are treated in MPIP (either inpatient or day clinic treatment). The participants are going to receive either tVNS or sham tVNS for a period of 6 weeks. Both tVNS and sham tVNS will be carried out as an adjuvant, i.e. in addition to the regular treatment of the participants. The intervention will take place three times a day from Monday to Friday. Each of the three daily sessions is going to last 30-60 minutes, depending on patient tolerance. The procedures for sham tVNS will be identical to tVNS, with the only exception that the sham tVNS will be performed with no current output. Participants and investigators are blinded to the assignment of participants to a study arm, except for the investigators responsible for the stimulation. The AddVNS study includes a wide range of assessments, which will be identical for both study arms. Psychophysiological measurements (pupillometry, respiratory rate, 3-channel ECG, photoplethysmography, electrogastrogram) will be conducted at baseline, after 3 weeks of intervention and at the end of the 6-week intervention (for a small number of patients (PILOT), with two additional psychophysiological measurements). Actigraphy will take place throughout the 6-week intervention. Two MRI scans are planned over the course of the AddVNS study; one scan is scheduled at baseline and one after the 6-week intervention. Participants will have a total of three venous blood samples taken over the course of the 6-week intervention. They will be asked to collect stool samples that will be used for microbiome analysis at baseline, after 3 weeks of intervention and at the end of the 6-week intervention. Participants will be also asked to fill out self-rating questionnaires (patient health questionnaire 9 and 15, Beck Depression Inventory II, Snaith-Hamilton-Pleasure-Scale, Questionnaire for complaints of cognitive disturbances) once a week, while two further questionnaires will be obtained at baseline and after the 6-week intervention (Rejection Sensitivity Questionnaire, World Health Organization Disability Assessment Schedule questionnaire). During a clinical interview at baseline, after 3 weeks of intervention and after 6 weeks of intervention, clinician-rating scales (Montgomery-Åsberg Depression Rating Scale, Hamilton Rating Scale for Depression with 21 items, Hamilton Rating Scale for Anxiety, Global Assessment of Functioning) will be obtained. Furthermore, a neuropsychological assessment is carried out at baseline and after the 6-week intervention, while a personality assessment will be performed at baseline. To assess the longitudinal effects of tVNS, we will ask the patients to fill out self-rating questionnaires (patient health questionnaire 9 and 15, Beck Depression Inventory II, Snaith-Hamilton-Pleasure-Scale, Questionnaire for complaints of cognitive disturbances, Rejection Sensitivity Questionnaire, World Health Organization Disability Assessment Schedule questionnaire) 6 and 12 weeks after the end of the intervention.

Safety considerations

No substances or medications are administered in the AddVNS study. Participants may also discontinue their participation at any time without providing reasons. Overall, the study is expected to impose a very low burden. Based on current knowledge, no health risks, suffering, pain, or lasting harm are anticipated. The risks and burdens are discussed individually and in detail below.

Burden and risks of transcutaneous auricular vagus nerve stimulation: tVNS is a safe, non-invasive procedure. Its safety and tolerability have been demonstrated in systematic reviews and a meta-analysis. Nonetheless, side effects may occur with tVNS, as with any procedure. The unwanted effects of tVNS are mostly mild, local reactions such as slight ear pain and skin redness at the stimulation site, or nonspecific symptoms such as mild headache, nausea, and dizziness. To our knowledge, no serious adverse events—particularly no serious cardiac events—have been reported in association with tVNS to date. The risk and intensity of adverse effects do not differ between tVNS and control groups.

Burden and risks of MRI: The MRI examinations of the neurocranium are conducted at intervals of approximately 6 weeks. There is no additional organizational burden for participants, as they are already receiving inpatient or day clinic treatment on site. The examinations pose only a very minor burden in the form of mild stress—partly due to the noise level (despite noise-canceling ear protectors) and the confined space of the MRI scanner, and partly due to the study situation itself. Relevant burdens are not expected if participants remain compliant during the examination. As with any examination, certain risks exist. In this case, all contraindications (especially ferromagnetic objects or residues in the body and severe claustrophobia) are ruled out during the study briefing, thereby reducing these risks to a negligible minimum. Throughout the entire MRI examination, medical staff are present and available to participants for questions, concerns, or any issues that arise.

Burden and risks of psychophysiological measurements: There are no reasonably expected burdens or risks associated with the psychophysiological measurements conducted during the study.

Burden and risks of blood sampling: The risks of peripheral venous blood sampling are very low. As with any breach of the skin barrier, there is a small risk of contamination or infection and thus a risk of complications; however, with proper technique and skin disinfection, this risk can be reduced to an absolute minimum. Since the blood draws in the AddVNS study are

aligned with clinically indicated and scheduled blood sampling during the patients' inpatient or day clinic treatment, no study-related additional venipunctures are planned. This does not create any additional risk for the study participants. The amount of blood drawn per session (~47 ml) represents a minor study-related burden for participants in the sense of a small blood loss (cumulative volume: ~141 ml). For comparison, a single blood donation involves the removal of 500 ml of venous blood at once. The blood draws spread over approximately eight weeks are comparatively small and allow the bone marrow sufficient time to regenerate.

Burden and risks of non-invasive stool sample collection

There are no reasonably expected burdens or risks associated with the non-invasive collection of stool samples.

Burden and risks of neuropsychology, personality assessment, and questionnaire-based psychometrics

There are no reasonably expected burdens or risks associated with the neuropsychology, personality assessment, and questionnaire-based psychometrics.

Follow-up

Two follow-up assessments are planned approximately 6 and 12 weeks after completion of the stimulation period. These follow-up visits consist exclusively of questionnaire-based evaluations. As no relevant burden or risk is associated with these assessments or with study participation overall, no additional clinical follow-up is planned in this context.

Should any clinically actionable pathological findings arise during the course of routine study procedures—specifically findings that can be interpreted with clinical certainty and that constitute incidental findings rather than research results—participants may provide consent to be recontacted. In such cases, participants will be informed about the incidental finding in accordance with institutional clinical procedures. No recontact will occur for findings lacking clinical certainty or for results derived solely from exploratory research analyses.

Data management and statistical analysis

Samples and data are double-coded (identification code) from the moment of collection and stored in this form in the biobank or research database at the Max Planck Institute of Psychiatry and within the Max Planck Society. They are linked to biomaterials and data already stored

there and jointly analyzed when necessary. All data analyses (including genetic analyses) are carried out using the numerical study code generated by the biobank.

Analyses of the study data and results will in some cases also be conducted in collaboration with other research groups. In such cases, all samples and data collected in the AddVNS study—including those obtained from the local biobanking project that are required to address the AddVNS research question (e.g., blood samples, molecular measurements, genetic analyses, data on illness, treatment and clinical course, socioeconomic background information, and psychometric/questionnaire data)—will be shared in pseudonymized form with scientific collaboration partners within and outside the European Union (EU), always contingent upon the written consent of the AddVNS participants.

Quality assurance

All study physicians involved in the AddVNS study regularly obtain and renew their Good Clinical Practice (GCP) training to ensure compliance with national and international standards for clinical research. The study is monitored by the internal study center along with local site monitoring structures, ensuring continuous oversight of protocol adherence, data quality, and participant safety. In addition, data protection consultation and oversight are provided through the Max Planck Society, ensuring full compliance with the General Data Protection Regulation (GDPR) and all institutional data security requirements.

Expected outcomes of the study

We expect the study to identify psychophysiological and imaging markers that differentiate the clinical trajectory of patients receiving active tVNS from those with sham. By integrating a multi-layer approach including, but not limited to, biological, psychological, and stimulation-related parameters, we anticipate to identifying promising biomarker constellations that predict individual trajectories and profiles. The multimodal dataset is also expected to provide novel insights into the neurobiological mechanisms underlying tVNS effects in depressive disorders. Ultimately, the findings may guide the development of personalized stimulation protocols and inform future mechanistic hypotheses.

Duration of the project

The study will run for three years, during which continuous recruitment and ongoing execution of all study procedures will take place.

Problems anticipated

Because functional unblinding is a well-known challenge in neurostimulation studies, we have anticipated this as a potential problem area and addressed it through a double-blind design, regular titration procedures in both groups, identical electrode placement for verum and sham conditions, and block-wise randomization. Owing to these measures and the overall study design, no immediate problems are anticipated.

Ethics

The AddVNS study has been reviewed by the appropriate institutional committee and has furthermore received full ethical approval from the Munich University's (LMU) ethics committee at the 18.03.2025. All study procedures were evaluated and confirmed to comply with the ethical standards of the institution and with applicable national and international guidelines.

Informed consent forms

For this study, the informed consent form (ICF) and participant information materials are provided solely in the local language (German). No English translation has been prepared, as translations are not required for local study approval by the LMU Ethics Committee. Importantly, producing a translation at this stage would result in an ICF version that has **not** been reviewed or approved by the local ethics committee and therefore **cannot** be provided as an approved document. The study involves only one participant group and does not include additional interventions that would necessitate separate consent documents; thus, a single German-language ICF is sufficient for this protocol.

Budget: institutional budget

Other support for the project: none

Collaboration with other scientists or research institutions: none

Curriculum Vitae of principal investigator

Prof. Dr. med. Peter Falkai – Leopoldina
https://www.leopoldina.org/mitgliederverzeichnis/mitglieder/member/Member/show/peter-falkai/

Other research activities of the investigators

Prof. Dr. med. Peter Falkai – Leopoldina
https://www.leopoldina.org/mitgliederverzeichnis/mitglieder/member/Member/show/peter-falkai/

References

1. Parente, J., et al., *Neural, Anti-Inflammatory, and Clinical Effects of Transauricular Vagus Nerve Stimulation in Major Depressive Disorder: A Systematic Review.* Int J Neuropsychopharmacol, 2024. **27**(3).
2. de Melo, P.S., et al., *A Mechanistic Analysis of the Neural Modulation of the Inflammatory System Through Vagus Nerve Stimulation: A Systematic Review and Meta-analysis.* Neuromodulation, 2024.
3. Austelle, C.W., et al., *A Comprehensive Review of Vagus Nerve Stimulation for Depression.* Neuromodulation, 2022. **25**(3): p. 309-315.
4. Liu, C.H., et al., *Neural networks and the anti-inflammatory effect of transcutaneous auricular vagus nerve stimulation in depression.* J Neuroinflammation, 2020. **17**(1): p. 54.
5. Rosso, P., et al., *Vagus nerve stimulation and Neurotrophins: a biological psychiatric perspective.* Neurosci Biobehav Rev, 2020. **113**: p. 338-353.
6. Wang, Y., et al., *Vagus nerve stimulation in brain diseases: Therapeutic applications and biological mechanisms.* Neurosci Biobehav Rev, 2021. **127**: p. 37-53.
7. Ma, J., et al., *Vagus nerve stimulation as a promising adjunctive treatment for ischemic stroke.* Neurochem Int, 2019. **131**: p. 104539.
8. Carreno, F.R. and A. Frazer, *Vagal Nerve Stimulation for Treatment-Resistant Depression.* Neurotherapeutics, 2017. **14**(3): p. 716-727.
9. Grimonprez, A., et al., *The antidepressant mechanism of action of vagus nerve stimulation: Evidence from preclinical studies.* Neurosci Biobehav Rev, 2015. **56**: p. 26-34.
